# The Arabidopsis Resistance-Like Gene *SNC1* Is Activated by Mutations in *SRFR1* and Contributes to Resistance to the Bacterial Effector AvrRps4

**DOI:** 10.1371/journal.ppat.1001172

**Published:** 2010-11-04

**Authors:** Sang Hee Kim, Fei Gao, Saikat Bhattacharjee, Joseph A. Adiasor, Ji Chul Nam, Walter Gassmann

**Affiliations:** 1 Division of Plant Sciences, Christopher S. Bond Life Sciences Center and Interdisciplinary Plant Group, University of Missouri, Columbia, Missouri, United States of America; 2 Department of Chemistry, University of Missouri, Columbia, Missouri, United States of America; 3 Division of Biological Sciences, University of Missouri, Columbia, Missouri, United States of America; Ohio State University, United States of America

## Abstract

The *SUPPRESSOR OF rps4-RLD1* (*SRFR1*) gene was identified based on enhanced AvrRps4-triggered resistance in the naturally susceptible Arabidopsis accession RLD. No other phenotypic effects were recorded, and the extent of *SRFR1* involvement in regulating effector-triggered immunity was unknown. Here we show that mutations in *SRFR1* in the accession Columbia-0 (Col-0) lead to severe stunting and constitutive expression of the defense gene *PR1*. These phenotypes were temperature-dependent. A cross between *srfr1-1* (RLD background) and *srfr1-4* (Col-0) showed that stunting was caused by a recessive locus in Col-0. Mapping and targeted crosses identified the Col-0-specific resistance gene *SNC1* as the locus that causes stunting. *SRFR1* was proposed to function as a transcriptional repressor, and *SNC1* is indeed overexpressed in *srfr1-4*. Interestingly, co-regulated genes in the *SNC1* cluster are also upregulated in the *srfr1-4 snc1-11* double mutant, indicating that the overexpression of *SNC1* is not a secondary effect of constitutive defense activation. In addition, a Col-0 *RPS4* mutant showed full susceptibility to bacteria expressing *avrRps4* at 24°C but not at 22°C, while RLD susceptibility was not temperature-dependent. The *rps4-2 snc1-11* double mutant showed increased, but not full, susceptibility at 22°C, indicating that additional cross-talk between resistance pathways may exist. Intriguingly, when transiently expressed in *Nicotiana benthamiana*, SRFR1, RPS4 and SNC1 are in a common protein complex in a cytoplasmic microsomal compartment. Our results highlight SRFR1 as a convergence point in at least a subset of TIR-NBS-LRR protein-mediated immunity in *Arabidopsis*. Based on the cross-talk evident from our results, they also suggest that reports of constitutive resistance phenotypes in Col-0 need to consider the possible involvement of *SNC1*.

## Author Summary

Plants, like humans, have an immune system to defend against disease. This immune system seeks out the presence of disease-causing microbes and other invaders by detecting non-plant molecules and proteins. Plants rely on this surveillance to activate an antimicrobial response of appropriate strength at the right time; as with humans, an overactive immune system can be harmful to plants. We study how plants achieve an appropriate balance, using genetics and the interaction between the reference plant *Arabidopsis thaliana* and the bacterial plant pathogen *Pseudomonas syringae*. So-called plant resistance proteins are important activators of immunity that directly or indirectly intercept foreign proteins deployed by pathogens. Resistance proteins are generally thought to be highly specific detectors that only respond to a single pathogen protein. However, while working with a negative regulator of plant immunity called SRFR1, we discovered a surprising level of cross-talk between different resistance proteins that becomes evident only under certain environmental conditions such as low temperature. We also show that SRFR1 and these resistance proteins bind to each other, possibly explaining the observed cross-talk. Our work thus highlights linkages between resistance pathways and provides insight into the molecular architecture of the plant innate immune response.

## Introduction

Plants possess a highly effective immune system that responds to conserved non-self molecular patterns, or to specific pathogen-derived molecules deployed to alter host defenses [1]–[3]. The latter response, called effector-triggered immunity (ETI), is largely mediated by resistance (R) proteins that directly or indirectly detect the presence of pathogen effectors [3], [4], although mechanistically overlap between ETI and the response to molecular patterns can be observed [5], [6]. ETI can lead to programmed cell death termed the hypersensitive response (HR) [7], [8]. In the case of resistance to some viral and hemi-biotrophic bacterial pathogens, it has been shown that the HR is not causally related to resistance [9]–[13]. Nevertheless, the plant immune response is deleterious to plant growth, normal development, and seed set even in the absence of HR, and therefore needs to be tightly controlled [14].

In order to explore the molecular mechanisms that negatively regulate ETI, we performed a suppressor screen for reactivated AvrRps4-triggered resistance in the naturally susceptible Arabidopsis (*Arabidopsis thaliana*) accession RLD [15]. This screen yielded two mutant alleles in *SUPPRESSOR OF rps4-RLD1* (*SRFR1*). Mutations in *srfr1* enhanced resistance of RLD specifically to *Pseudomonas syringae* pv. tomato strain DC3000 (DC3000) expressing *avrRps4*, while susceptibility to the virulent strain DC3000 was unchanged [15]. Apart from re-establishing a certain level of resistance to *avrRps4*, no other marked phenotype was noted.

*RPS4* encodes an R protein of the Toll/Interleukin-1 receptor (TIR) - nucleotide binding site (NBS) - leucine-rich repeat (LRR) (TNL) class [16], and was found to require the defense regulator *EDS1* to trigger immunity [17]. This is in contrast to the coiled-coil (CC) -NBS-LRR (CNL) R proteins RPS2, RPM1 and RPS5, which require the defense gene *NDR1* [17]. Combining mutations in *SRFR1* and the CNL pathway genes *RPM1*, *RPS2* or *NDR1* did not measurably alter the susceptibility to the cognate effector genes. The partial resistance to *avrRps4* in *srfr1* mutants required *EDS1* [15], [18]. In addition, mutations in *RPS6*, another TNL gene that requires *EDS1* [12], led to susceptibility to DC3000(*hopA1*) that was diminished in *srfr1-1 rps6-1* double mutants [19].

Taken together, these data indicated that SRFR1 function is closely associated with the *EDS1* resistance pathway. Here we show that a mutation in *SRFR1* in the accession Columbia-0 (Col-0), *srfr1-4*, activates the Col-0 specific and *EDS1*-dependent *R*-like gene *SNC1*, consistent with the genetic function of *SRFR1* as a negative regulator of *R* gene-mediated resistance. Activation of constitutive defenses in *srfr1-4* was temperature-dependent. In addition, *RPS4* and *SNC1* contributed redundantly to susceptibility to DC3000(*avrRps4*) in Col-0 at 22°C, whereas at 24°C *RPS4* activity was the sole determinant of resistance. Interestingly, SRFR1 interacted with both RPS4 and SNC1. Our data thus provide evidence for cross-talk between these TNL pathways that converge on SRFR1, suggesting that SRFR1 may have a general function in regulating TNL protein signal output.

## Results

### A mutation in *SRFR1* in Col-0 causes abnormal growth

**Figure 1:**
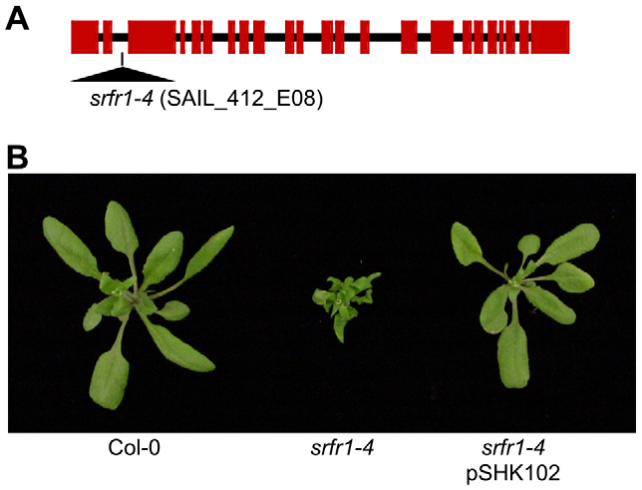
A mutation in *SRFR1* causes severe stunting in Col-0. (A) Schematic gene structure of *SRFR1* (At4g37460), with exons shown as boxes and introns as lines. The T-DNA insertion site in the second intron in *srfr1-4* (SAIL_412_E08) is indicated. (B) Growth phenotype of *srfr1-4* and complementation with a genomic copy of *SRFR1* in transgenic plants.

We previously had isolated the mutant alleles *srfr1-1* and *srfr1-2* from the Arabidopsis accession RLD [15]. Apart from enhanced resistance to DC3000(*avrRps4*), they did not display marked phenotypes. To further investigate the function of SRFR1, we aimed at isolating T-DNA tagged lines of *SRFR1* in the accession Col-0 [20], [21]. Out of four lines, one did not germinate (SALK_106212), and one was untagged (SALK_095440). We could verify a T-DNA insertion far upstream of the open reading frame in SALK_039199, without causing an apparent phenotype. Interestingly, the fourth line, SAIL_412_E08 with a T-DNA insertion in the second intron of *SRFR1* (Figure 1A), showed pronounced stunting (Figure 1B) in one-fourth of plants (22 out of 97 plants; χ^2^ = 0.28). Genotyping showed that the T-DNA insertion in *SRFR1* segregated in the original seed stock, and that stunted plants were invariably homozygous for the T-DNA insertion. Reverse transcription (RT) PCR showed that no *srfr1* mRNA was detected with primers on either side of the insertion (Figure S1). A low level of *srfr1* mRNA could be detected with primers located 3′ of the T-DNA insertion, but this mRNA contained the T-DNA (Figure S1), indicating that *srfr1-4* mRNA does not encode functional protein. Consistent with this, Li and co-workers recently showed that no SRFR1 protein can be detected in this knock-out line [22]. We named this line *srfr1-4*.

Subsequently, we back-crossed *srfr1-4* to Col-0. The stunted phenotype co-segregated with homozygosity of the *srfr1-4* T-DNA tagged allele in F2 plants (Table 1). To prove that the phenotype originated from the *srfr1-4* allele, we transformed healthy heterozygous *srfr1-4* plants with pSHK102 containing a genomic clone of *SRFR1* [18], and by scoring for antibiotic resistance selected 5 single-locus homozygous transgenic *SRFR1* T3 lines that contained at least one copy of the *srfr1-4* T-DNA allele based on genotyping. Because the transgenic copy of *SRFR1* prevented us from determining whether these T3 lines were homozygous or heterozygous for the *srfr1-4* allele, we tested whether *srfr1-4* segregated in the next generation by genotyping 15 progeny for each line. Three of the 5 lines were shown in this way to be homozygous for the *srfr1-4* allele, and the transgenic copy of *SRFR1* reversed the stunted phenotype in each case (Figure 1B). We concluded that the stunted growth phenotype is caused by the T-DNA insertion in *SRFR1*.

**Table 1:** The stunted phenotype co-segregates with the *srfr1-4* allele in a backcross to Col-0.

Generation	Phenotype	n	χ^2^	Genotype	n
F1	normal	4		*SRFR1/srfr1-4*	4
F2	normal	57	0.071 (*P*>0.7)^a^	*SRFR1/SRFR1*	20
				*SRFR1/srfr1-4*	37
	stunted	18		*srfr1-4/srfr1-4*	18

a χ^2^ value for the expected ratio of 3 normal : 1 stunted progeny.

### Genetics of *srfr1*-mediated stunting

The stunted *srfr1-4* phenotype was in marked contrast to the normal phenotype of *srfr1-1* and *srfr1-2* plants. To determine whether the specific allele of *SRFR1* or the Col-0 genetic background causes the severe phenotype of *srfr1-4*, we first reexamined more closely F3 families of important break-point plants retained from the *SRFR1* mapping populations. Plants in these F3 families were generated by crossing *srfr1-1* or *srfr1-2* (RLD background) to the SAIL *RPS4* T-DNA knockout line *rps4-1* (Col-0 background) [15], [18] and were progeny of F2 plants selected for resistance to DC3000(*avrRps4*). They were therefore homozygous for *srfr1-1* or *srfr1-2*, with varying degrees of Col-0 background. Two out of 4 *srfr1-1* and 2 out of 6 *srfr1-2* F3 families contained no individuals with abnormal growth phenotypes. However, the remaining F3 families gave rise to plants with phenotypes similar to *srfr1-4*. The combined total number of stunted plants in these families was 20 out of 107 plants, consistent with the segregation of a single recessive gene in these populations (χ^2^ = 2.43, *P*>0.1). We concluded that most likely the mutant alleles *srfr1-1* and *srfr1-2* also induce stunting in the Col-0 background and that Col-0 possesses a recessive genetic modifier that alters the *srfr1* phenotype.

We tested these predictions directly by out-crossing *srfr1-4* to RLD and *srfr1-1*. In the cross to *srfr1-1*, 14 out of 46 plants were stunted, consistent with both *srfr1-1* and *srfr1-4* causing stunting and the segregation of a recessive gene (χ^2^ = 0.45, *P*>0.5). In the cross to RLD, segregation of the stunted phenotype in the F2 generation was explained by two recessive genes, and genotyping showed that while all stunted plants were homozygous *srfr1-4*, not all *srfr1-4/srfr1-4* plants were automatically stunted (Table 2). In this cross, stunted F2 plants were also selected to determine a rough map position for the presumptive Col-0 modifier gene. This mapping placed the Col-0 modifier gene onto chromosome 4 (Table 3). Interestingly, in addition to the bottom of chromosome 4 where *SRFR1* is located, individual break-point plants identified a map position towards the top of chromosome 4 between markers ciw6 and CH42 for the Col-0 modifier gene.

**Table 2:** A second recessive Col-0 allele is required for stunting in the cross RLD×*srfr1-4*.

Generation	Phenotype	n	χ^2^	Genotype^b^	n
F1	normal	6		*SRFR1/srfr1-4*	6
F2	normal	435	2.28 (*P*>0.1)^a^	*SRFR1/SRFR1*	12
				*SRFR1/srfr1-4*	27
				*srfr1-4/srfr1-4*	10
	stunted	38		*srfr1-4/srfr1-4*	38

a χ^2^ value for the expected ratio of 15 normal : 1 stunted progeny.

b Only 49 of the 435 normal F2 plants were genotyped.

**Table 3:** Mapping of the Col-0 modifier gene in stunted F2 plants from the cross RLD×*srfr1-4*.

Marker (Mb)^a^	Recombinant Chromosomes	Total Number of Chromosomes	Recombination Frequency (%)
nga8 (5.6)	21	86	24
DET1.2 (6.3)	12	84	14
ciw6 (7.9)	2	58	3
CH42 (10.2)	2	86	2
20B4L-1.6 (11.1)	1	86	1
nga1139 (16.4)	0	52	0
nga1107 (18.1)	4	80	5

a The position for *SNC1* on chromosome 4 is at 9.5 Mb, and that for *SRFR1* at 17.6 Mb. Markers that did not show linkage with the stunted phenotype were nga63 and nga280 (chromosome 1), nga168 (chromosome 2), nga162 and nga6 (chromosome 3), and nga225 and nga139 (chromosome 5).

### *srfr1-4* has constitutively activated defenses caused by *SNC1*

**Figure 2:**
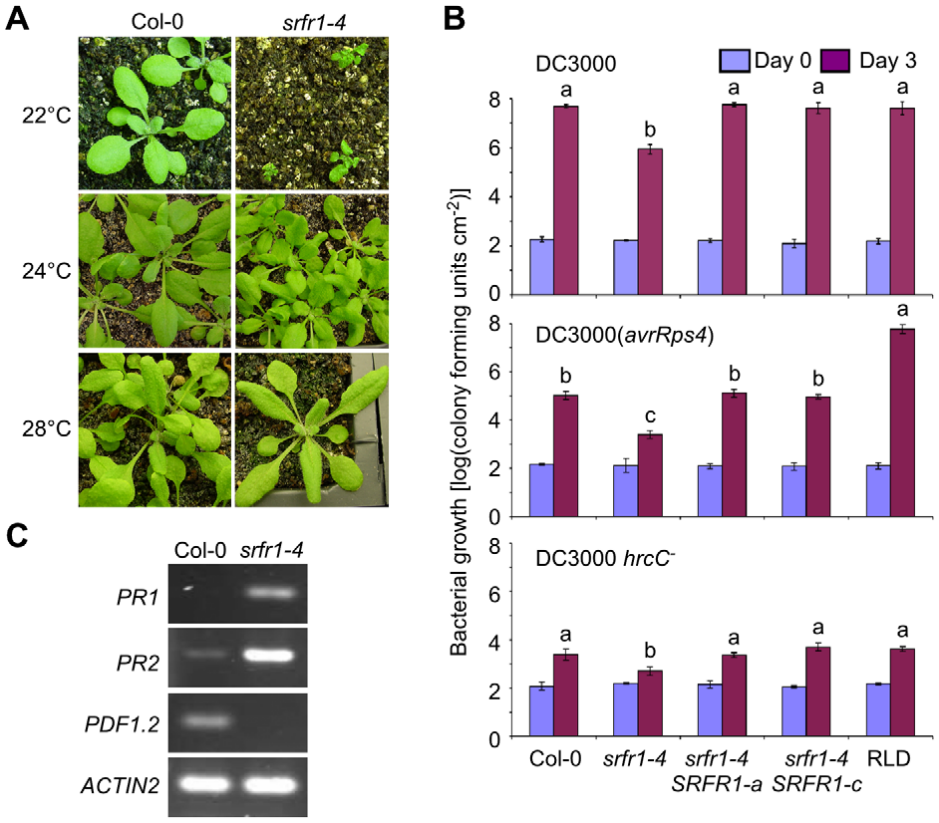
The growth phenotype of *srfr1-4* is temperature-dependent and accompanied by constitutive activation of defenses. (A) Growth phenotype of wild type Col-0 (left column) and *srfr1-4* (right column) at 22°C (top row), 24°C (middle) and 28°C (bottom). (B) The *srfr1-4* mutation enhances both basal defenses and AvrRps4-triggered immunity in Col-0. *In planta* bacterial growth at 24°C of DC3000 (top), DC3000(*avrRps4*) (middle) and DC3000 *hrcC^−^* on day 0 (blue bars) and day 3 (purple bars) after inoculation of the indicated plant lines with bacteria at 5×10^4^ colony-forming units (cfu) per ml. Two independent transgenic *srfr1-4* lines complemented with a genomic copy of *SRFR1* are shown. Values represent averages of cfu/cm^2^ leaf tissue from triplicate samples, and error bars denote standard deviation. Values labeled with different letters show significant differences as determined by Student's t-test (*P*<0.05, n = 3) on day 3. This experiment was repeated twice with similar results. (C) Altered defense gene mRNA levels in *srfr1-4* at 22°C. Analysis of indicated transcripts in Col-0 and *srfr1-4* by RT-PCR using 27 PCR cycles. *ACTIN2* was used as an internal control.

The map position for the modifier gene contained the Col-0-specific TNL *R* gene homolog *SNC1*, which was originally identified through a point mutation that autoactivates the SNC1 protein and constitutively induces *PR* genes even in the *npr1* mutant line [23]. Additional work showed that wild-type *SNC1* is easily autoactivated when expression of *SNC1* is misregulated [24]. For example, mutations in *BON1*, a member of the copine gene family encoding a plasma membrane-localized putative calcium-dependent phospholipid-binding protein [25], [26], lead to higher *SNC1* expression levels, constitutive defense responses and reduced plant growth [27]. When the Col *bon1-1* mutant was outcrossed to other Arabidopsis accessions, it was found that the wild-type *SNC1* gene from Col-0 behaved as a recessive locus that causes stunting [27]. Our segregation data also indicated that the Col-0 modifier was recessive (Table 2). We therefore tested additional phenotypes displayed by *bon1-1* plants, such as temperature dependence of constitutive defense activation and growth phenotypes. The stunted phenotype in *srfr1-4* was severe at 22°C, but was intermediate at 24°C and absent at 28°C (Figure 2A), reminiscent of the Arabidopsis *bon1-1* mutant phenotype.

In *srfr1-1* and *srfr1-2* plants, resistance to DC3000(*avrRps4*) was enhanced, but remained unchanged to virulent DC3000, and plant growth was normal [15], [18]. Interestingly, the *srfr1-4* mutants were resistant not only to avirulent DC3000(*avrRps4*), but also to virulent DC3000 and non-pathogenic DC3000 *hrcC^−^* (Figure 2B). The *srfr1-4* line showed approximately 50-fold lower DC3000 and DC3000(*avrRps4*) growth than wild type Col-0, whereas the growth of DC3000 *hrcC^−^* in *srfr1-4* was about 10-fold less than in Col-0, suggesting that mutations in *SRFR1* in Col-0 increased basal defenses at 24°C that were additive to AvrRps4-triggered immunity (Figure 2B). Complemented *srfr1-4* lines did not show either enhanced resistance phenotype (Figure 2B). We could not test bacterial growth at 22°C because *srfr1-4* plants were severely stunted at this temperature. However, consistent with an upregulation of salicylic acid (SA)-based defenses, *PR1* and *PR2* mRNA levels were upregulated and *PDF1.2* levels down-regulated in *srfr1-4* at 22°C (Figure 2C).

**Figure 3:**
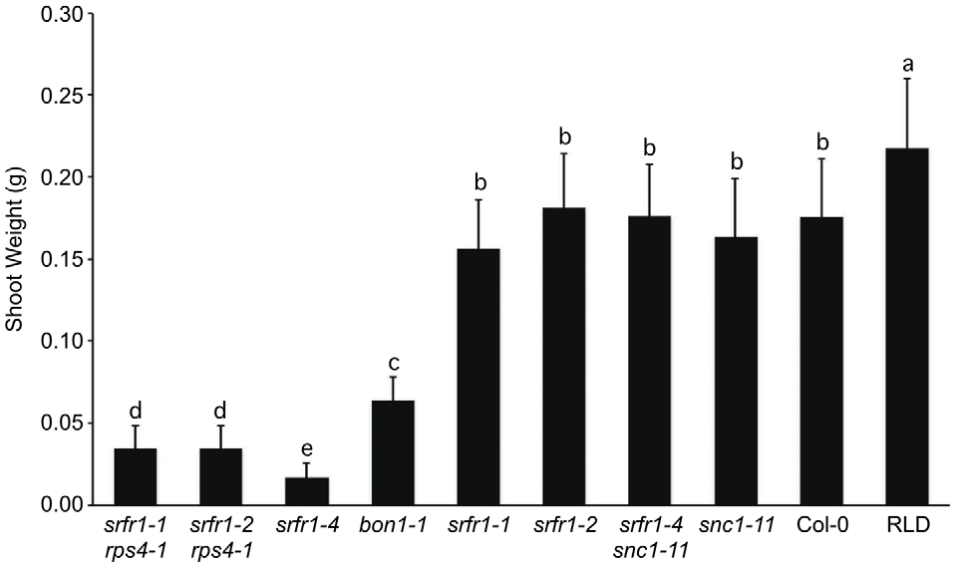
Stunting of *srfr1-4* as measured by shoot weight is reversed by *snc1-11*. The indicated plant lines were grown for three weeks in a greenhouse at 22°C with a 16 h light/8 h dark cycle. Values represent average shoot weights of 40 to 70 plants for each line, and error bars denote standard deviation. Values labeled with different letters show significant differences as determined by Student's t-test (*P*<0.05). This experiment was repeated once with similar results.

Characterization of the *srfr1-4* phenotype and mapping therefore strongly suggested that the Col-0 modifier is *SNC1*. To test this directly, we crossed *srfr1-4* to *snc1-11*, a T-DNA insertion allele in the first exon of *SNC1* [27]. In the F2 population, the number of stunted plants was consistent with the segregation of two recessive loci (*srfr1-4* and wild-type *SNC1*) (Table 4). All of the stunted plants were homozygous for the *srfr1-4* allele and the wild-type *SNC1* allele. In contrast, all plants of normal stature that were homozygous for the *srfr1-4* T-DNA allele possessed at least one copy of the *snc1-11* T-DNA allele (Table 4). Therefore, the stunted phenotype of *srfr1-4* plants requires two copies of *SNC1* in Col-0, analogous to the phenotype of *bon1-1* plants [27]. We quantified the effect of mutations in *SRFR1* on plant growth by measuring the shoot weight of *srfr1* mutants in Col-0 and RLD (Figure 3). Shoot weights were close to normal in the original *srfr1-1* and *srfr1-2* plants compared to wild-type RLD. Mutations in *srfr1* caused severe reductions in shoot weight in the Col-0 background that were completely reversed by introgressing *snc1-11*. Interestingly, the shoot weight of *srfr1 SNC1* plants was more strongly reduced than in *bon1-1* plants (Figure 3), indicating that perhaps *SRFR1* functions downstream of additional *R* genes apart from regulating *SNC1*. Together with the negative regulation in AvrRps4- and HopA1-triggered immunity, these results show that SRFR1 is a negative regulator of plant immune responses of broader specificity than originally described.

**Table 4:** Two copies of *SNC1* are required for stunting in F2 plants from the cross *snc1-11*×*srfr1-4*.

Generation	Phenotype	n	χ^2^	Genotype^b^	n
F1	normal	5		*SRFR1/srfr1-4 SNC1/snc1-11*	5
F2	normal	202	0.31 (*P*>0.5)^a^	n.d.	72
				*SRFR1/SRFR1*	36
				*SRFR1/srfr1-4*	75
				*srfr1-4/srfr1-4 SNC1/snc1-11*	14
				*srfr1-4/srfr1-4 snc1-11/snc1-11*	5
	stunted	16		*srfr1-4/srfr1-4 SNC1/SNC1*	16

a χ^2^ value for the expected ratio of 15 normal : 1 stunted progeny.

b Only 130 of the 202 normal F2 plants were genotyped at the *SRFR1* locus. Only homozygous *srfr1-4* plants were further genotyped at the *SNC1* locus.

### The LRR domain is deleted in SNC1-RLD

**Figure 4:**
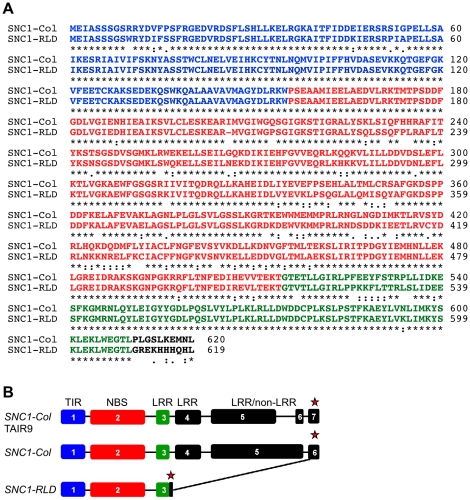
*SNC1-RLD* encodes a truncated TNL protein. (A) Alignment of deduced amino acid sequences of SNC1-Col (top) and SNC1-RLD (bottom) using the EBI-ClustalW tool (http://www.ebi.ac.uk/Tools/clustalw/) [54]. Identical amino acids are indicated by asterisks. Colons and semi-colons show conserved substitutions and semi-conserved substitutions, respectively. Characters in blue, red and green show the amino acids corresponding to exon 1, exon 2 and exon 3, respectively. (B) *SNC1* gene model as experimentally verified by reverse transcription PCR and 3′-RACE from Col-0 (middle) and RLD (bottom) compared with the TAIR9 gene model (top). Exons are indicated by boxes, introns by lines, and stop codons by red asterisks.

Previous studies had suggested that the readily autoactivatable SNC1 is limited to the Col-0 accession, but these studies had not included RLD [27]. We therefore sequenced the likely RLD ortholog of *SNC1* in RLD to determine the molecular basis for the very different phenotypes of Col-0 and RLD *srfr1* mutants. At the 5′-end, *SNC1*-specific primers consistently amplified a sequence with high overall similarity to *SNC1-Col* (Figure 4A and 4B). *SNC1*-specific primers designed to amplify the complete *SNC1* gene or the 3′-half of *SNC1* failed to result in a unique RLD product. This reflected the very duplicated nature of the 3′-half of *SNC1* in Col-0. Whole sections of the gene are not only duplicated within *SNC1* with 100% sequence identity, but are also found in linked family members [28]. We were not able to experimentally determine unequivocally which genomic PCR product from the 3′-end was physically linked to the 5′-end of *SNC1-RLD*.

We therefore determined the *SNC1* mRNA sequence from RLD using a combination of 3′-Rapid Amplification of cDNA Ends (3′-RACE) and RT-PCR. As shown in Figure 4A, the open reading frame of *SNC1-RLD* predicted a protein of 619 amino acids, including a TIR and NBS domain but only a partial LRR domain. The predicted amino acid sequence identity between SNC1-Col and SNC1-RLD within the first three exons was 87%. However, our *SNC1-RLD* cDNA sequence was missing the fourth and fifth exons, leading to an in-frame stop codon at position 620 (Figure 4B). Interestingly, in the *SNC1-RLD* cDNA the very 3′-end of the open-reading frame and the 3′-untranslated region showed high nucleotide sequence identity with the corresponding region in *SNC1-Col*. Because we only obtained cDNA sequence of *SNC1-RLD* at the 3′-end, we could not determine whether the 3′-end of the *SNC1-RLD* coding sequence is interrupted by introns. We also obtained RT-PCR products from Col-0. These indicated that in contrast to the annotation of *SNC1* in TAIR, we did not find evidence for the splicing of intron 5, which does not contain in-frame stop codons (Figure 4B). This alternative *SNC1* transcript encoded a SNC1 protein of 1404 amino acids rather than the annotated 1301 amino acids. Taken together, sequencing of the RLD *SNC1* ortholog provided evidence for polymorphisms at the 5′-end and major alterations in the 3′-half of the gene compared to Col-0, consistent with the fact that RLD does not have a *SNC1* ortholog that triggers stunted growth in the absence of *SRFR1*.

### Morphologically normal *srfr1-4 snc-11* double mutants possess primed defenses

Activation of *SNC1*, either by intragenic autoactivating mutations [23] or by mutations in negative regulators of *SNC1* such as *BON1* [27], leads to constitutively enhanced resistance. Consistent with this and the constitutive expression of *PR* genes in *srfr1-4* (Figure 2C), we observed with *in planta* bacterial growth assays increased resistance of *srfr1-4* to DC3000(*avrRps4*) and to virulent DC3000 (Figure 2B). The latter shows that *srfr1-4* plants possess elevated basal resistance that is independent of particular avirulence genes. To test if enhanced basal resistance in *srfr1-4*, like stunted growth, is fully dependent on *SNC1*, we performed *in planta* bacterial growth assays at varying temperatures. As noted before, we were not able to infiltrate *srfr1-4* plants at 22°C because of the severe growth phenotype.

**Figure 5:**
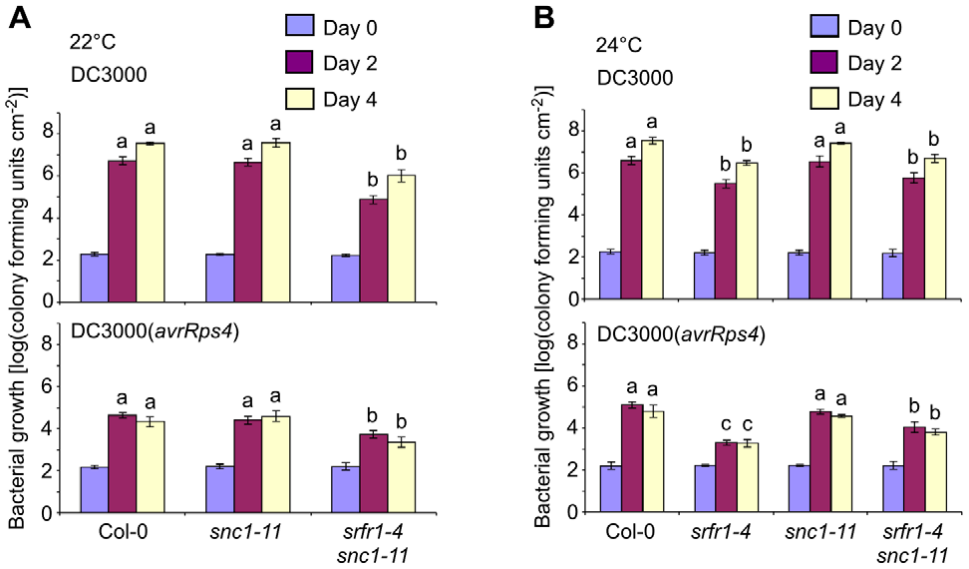
Phenotypically normal *srfr1-4 snc1-11* double mutants show enhanced basal defense and AvrRps4-triggered immunity. *In planta* bacterial growth was measured in the indicated plant lines on day 0 (blue bars), day 2 (purple) and day 4 (yellow) after inoculation with DC3000 (top) and DC3000(*avrRps4*) (bottom) at 5×10^4^ cfu/ml at 22°C (A) and 24°C (B). Values represent averages of cfu/cm^2^ leaf tissue from triplicate samples, and error bars denote standard deviation. Values labeled with different letters show significant differences at the indicated days as determined by Student's t-test (*P*<0.05, n = 3). This experiment was repeated twice with similar results.

At both 22°C and 24°C, the growth of DC3000 and DC3000(*avrRps4*) was reduced in *srfr1-4 snc1-11* compared to growth in wild type Col-0, even though the growth of DC3000(*avrRps4*) in *srfr1-4 snc1-11* was slightly higher than that in *srfr1-4* at 24°C (Figure 5A and 5B). This remnant enhanced basal resistance in *srfr1-4 snc1-11* plants may be related to the induced defense gene mRNA levels observed in RLD *srfr1-1* and *srfr1-2* plants, although the latter plants do not show enhanced basal resistance [18], [29]. These results demonstrate that although the stunted phenotype of *srfr1-4* at 22°C and 24°C is fully mediated by *SNC1*, enhanced basal resistance at these temperatures in *srfr1-4* is not entirely mediated by *SNC1*. At 28°C, both basal and AvrRps4-triggered resistance were abolished in *srfr1-4* and *srfr1-4 snc1-11* plants (Figure S2A). In addition, AvrRps4-triggered resistance was also abolished in wild-type Col-0, confirming previous results [30], and in *snc1-11* plants (Figure S2A). Consistent with normal growth and absence of resistance at 28°C, *SNC1* and *PR1* expression were not elevated in *srfr1-4* or *srfr1-4 snc1-11* plants (Figure S2B).

### Altered expression levels of defense-related genes in *srfr1-4* and *srfr1-4 snc1-11*

**Figure 6:**
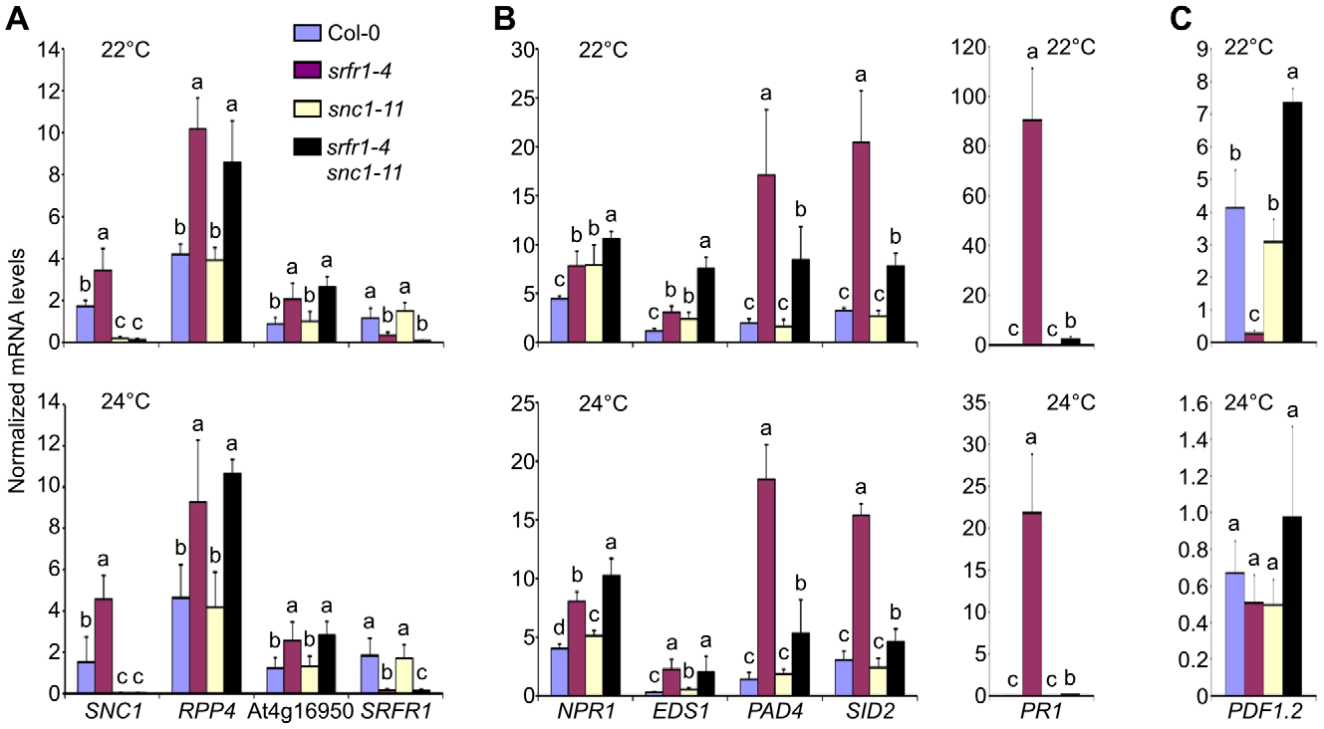
Transcript levels of defense-related genes are altered in *srfr1-4* and *srfr1-4 snc1-11*. (A) Transcript levels of the co-regulated *R* genes *SNC1*, *RPP4* and At4g16950. (B) Transcript levels of *NPR1*, *EDS1*, *PAD4* and *SID2* (left) and *PR1* (right). (C) Transcript levels of *PDF1.2*. Transcript levels were measured by qPCR in Col-0 (blue bars), *srfr1-4* (purple), *snc1-11* (yellow) and *srfr1-4 snc1-11* (black) at 22°C (top) and 24°C (bottom), and were normalized using *SAND* gene (At2g28390) mRNA levels as an internal control. Values represent averages from four biological replicates, and error bars denote standard deviation. Different letters denote significant differences between values calculated by Student's t-test (*P*<0.05, n = 4). This experiment was repeated once with similar results.

Previously, we showed that several defense-related genes were up-regulated in RLD *srfr1* mutants, supporting our hypothesis that SRFR1 may function as a repressor in plant innate immunity by negatively regulating defense gene expression levels [29]. The growth and constitutive defense phenotypes of *srfr1-4* at 22°C and 24°C prompted us to quantify defense-related gene mRNA levels in *srfr1-4* at these temperatures using quantitative reverse transcription real-time PCR (qPCR), and to determine whether all changes in expression in *srfr1-4* can be attributed to *SNC1*. As expected, *SNC1* transcript levels were higher in *srfr1-4* than in Col-0 at 22°C and 24°C, as were those of *RPP4* and At4g16950 (Figure 6A), two TNL genes in the *SNC1* cluster that are co-regulated with *SNC1* [31]. Interestingly, *RPP4* and At4g16950 expression levels were higher also in the *srfr1-4 snc1-11* double mutant (Figure 6A), showing that higher mRNA levels of these genes is not an indirect effect of *SNC1* activation. Similarly, we observed increased mRNA levels of the CNL *R* gene *RPS2*, and to a lesser extent of *RPM1*, in *srfr1-4* and *srfr1-4 snc1-11* plants at both 22°C (Figure S3A) and 24°C (Figure S3B), indicating that upregulation of *R* genes by mutations in *SRFR1* is not limited to TNL genes in Col-0. In contrast to *SNC1-RLD*, upregulation of *RPM1* and *RPS2* was not observed in the RLD mutant *srfr1-1* (Figure S3C), possibly reflecting the presence of additional accession-specific *SNC1*-like genes in Col-0 [32] that may lead to enhanced expression of CNL genes.

SA-dependent defense related gene mRNA levels were also higher in *srfr1-4* than in wild-type at 22°C and 24°C (Figure 6B). Unlike for TNL and CNL genes, these expression levels were reduced in *srfr1-4 snc1-11* compared to *srfr1-4* to varying degrees, although they were still higher than in wild-type (Figure 6B). Interestingly, *NPR1* and *EDS1* mRNA levels in the double *srfr1-4 snc1-11* mutant showed additive increases compared to the wild-type and single mutants at 22°C (Figure 6B). In contrast, mRNA levels of *PDF1.2*, a defensin gene whose expression is under negative regulation by the JA-responsive transcription factor JIN1 [33], was strongly repressed at 22°C in *srfr1-4* but induced in *srfr1-4 snc1-11* plants compared to wild-type. *PDF1.2* expression levels were not significantly different among the genotypes at 24°C (Figure 6C). These results point towards complex modular control of defense gene expression that is influenced by a combination of *SRFR1*, *SNC1* and temperature to varying proportions.

### Cross-talk between *RPS4* and *SNC1* in AvrRps4-triggered immunity

**Figure 7:**
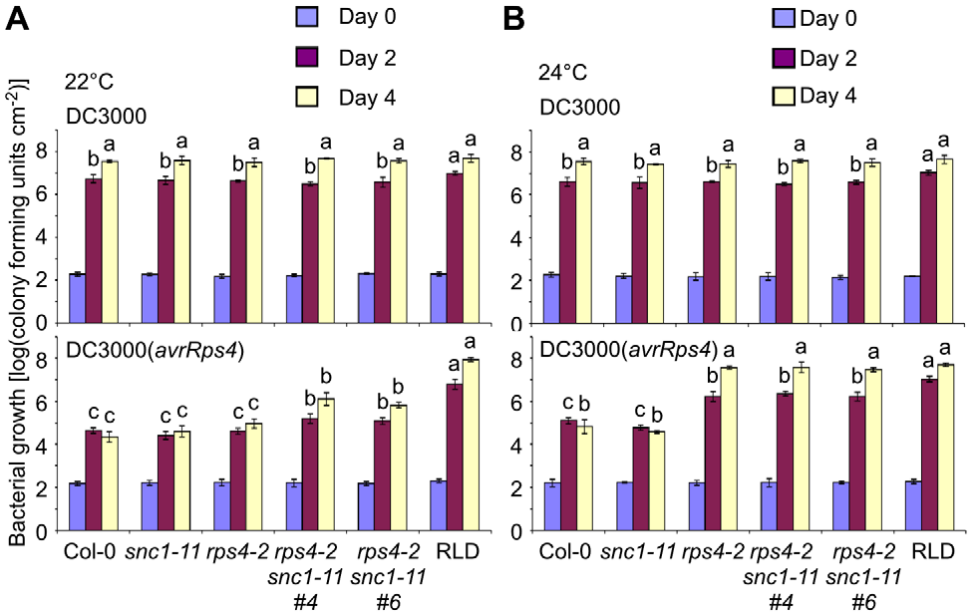
*RPS4* and *SNC1* contribute redundantly to AvrRps4-triggered resistance. *In planta* bacterial growth was measured in Col-0, *snc1-11*, *rps4-2*, two independent *snc1-11 rps4-2* lines and RLD on day 0 (blue bars), day 2 (purple) and day 4 (yellow) after inoculation with DC3000 (top) and DC3000(*avrRps4*) (bottom) at 5×10^4^ cfu/ml at 22°C (A) and 24°C (B). Values represent averages of cfu/cm^2^ leaf tissue from triplicate samples, and error bars denote standard deviation. Values labeled with different letters show significant differences at the indicated days as determined by Student's t-test (*P*<0.05, n = 3). This experiment was repeated three times with similar results.

The Arabidopsis accession RLD carries a natural mutation in *RPS4* and is fully susceptible to DC3000(*avrRps4*) [34], [35]. In addition, introduction of *RPS4* from Col-0 or L*er* into RLD is sufficient to provide full resistance to DC3000(*avrRps4*) when compared to Col-0 and L*er* [16], [35]. We also observed susceptibility of *rps4-1*, an *RPS4* T-DNA allele in the Col-0 background, under our conditions that were used to map *SRFR1* [15]. However, it was reported that *rps4-2*, a second *RPS4* T-DNA allele in the Col-0 background, was only slightly more susceptible to DC3000(*avrRps4*) [36]. Based on the accession-specific presence of *SNC1* in Col-0, the temperature-dependent *srfr1-4* phenotype and the fact that *SRFR1* was identified in a screen for enhanced DC3000(*avrRps4*) resistance in RLD, we speculated that the *rps4-2* phenotype might be temperature-dependent. Indeed, when directly comparing plants grown in identical growth chambers at 22°C or 24°C, we observed a strong temperature dependence: *rps4-2* plants were as resistant to DC3000(*avrRps4*) as Col-0 at 22°C, while at 24°C they were as susceptible as Col-0 treated with virulent DC3000 and as susceptible as RLD treated with either strain (Figure 7).

Given the effect of temperature, we next tested whether *SNC1* interferes with the susceptible phenotype at 22°C. Interestingly, *rps4-2 snc1-11* double mutants displayed approximately 30-fold increased bacterial growth of DC3000(*avrRps4*) compared to Col-0 or *rps4-2* at 22°C (Figure 7A), suggesting that *SNC1* in the absence of *RPS4* contributes to AvrRps4-triggered immunity at 22°C in Col-0. However, susceptibility of *rps4-2 snc1-11* to DC3000(*avrRps4*) was not complete compared to Col-0 treated with virulent DC3000 or to RLD treated with either strain, indicating that additional factors interfere with *rps4*-caused susceptibility (Figure 7A). No significant difference of DC3000(*avrRps4*) growth in *rps4-2* and *rps4-2 snc1-11* was observed at 24°C, reflecting full susceptibility of *rps4-2* to DC3000(*avrRps4*) at this temperature (Figure 7B). Recently, *RRS1* was shown to be involved in DC3000(*avrRps4*)-mediated resistance [37], [38]. However, we observed no temperature-dependent resistance to DC3000(*avrRps4*) in the Ws-0 mutants *rps4-21* and *rrs1-1* (Figure S4). As was observed before, mutations in either *RPS4* or *RRS1* had equal effects on DC3000(*avrRps4*) susceptibility, which was qualitatively different from the redundancy between *SNC1* and *RPS4* (Figure 7). Interestingly, as reported before [38], we reproducibly observed approximately 10-fold higher growth of DC3000 compared to DC3000(*avrRps4*) in the single *rps4-21* and *rrs1-1* mutants and the double mutant, indicating that additional layers of resistance exist.

### SNC1 and RPS4 interact with SRFR1

The redundancy between *RPS4* and *SNC1* suggests that they function in parallel to provide resistance to DC3000(*avrRps4*) at 22°C. We speculated that this cross-talk between two R proteins might occur if both interact with proteins in a common complex. Perturbation of this complex by an effector could trigger one or the other R protein, and both need to be absent to observe susceptibility. Based on the results presented here, we reasoned that SRFR1 might be a common interaction partner of RPS4 and SNC1. In the past, transient expression of SRFR1 in *Nicotiana benthamiana* led to variable protein expression levels and required a silencing inhibitor for detectable expression [18]. We therefore generated stable transgenic *N. benthamiana* plants expressing HA-SRFR1 encoded by a genomic clone driven by the native Arabidopsis *SRFR1* promoter. We first determined the functionality of this genomic *HA-SRFR1* construct in Arabidopsis by testing for complementation of the stunted *srfr1-4* phenotype. Transgenic plants expressing *HA-SRFR1* in the *srfr1-4* background showed normal growth and development (Figure S5A). Immunoblot analysis detected the expression of the transgene product in these transgenic plants (Figure S5A). HA-SRFR1 in these plants localized to microsomal and nuclear fractions (Figure S5B). This localization was consistent with the nuclear and punctate cytoplasmic localization of GFP-SRFR1 transiently expressed in *N. benthamiana* [18].

**Figure 8:**
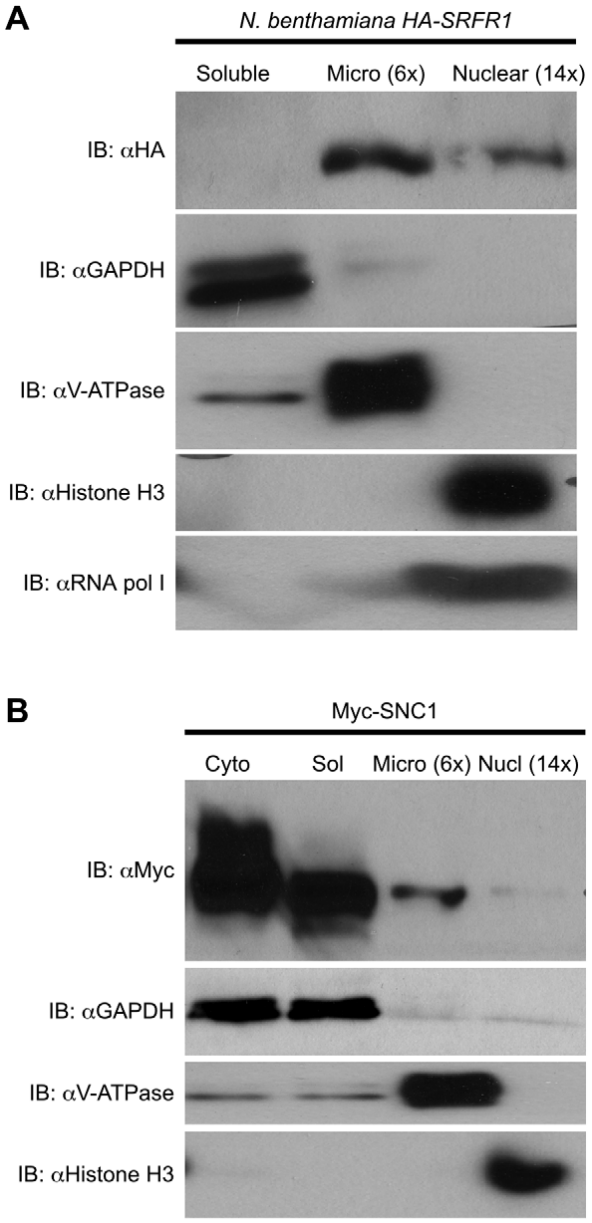
Localization of SRFR1 and SNC1 expressed in *N. benthamiana*. (A) HA-SRFR1 stably expressed in transgenic *N. benthamiana* localizes to the cytoplasmic microsomal and nuclear fractions. (B) Transiently expressed Myc-SNC1 localizes predominantly to the soluble cytoplasmic fraction, with detectable amounts in the microsomes and nuclei. In (A) and (B), the microsomal and nuclear fractions are 6 and 14 times concentrated, respectively, compared to the soluble fraction. The degree of fraction enrichment was determined using antibodies against marker proteins (anti-histone H3 and anti-RNA polymerase I subunit, nucleus; anti-GAPDH, cytoplasmic soluble; and anti-V-ATPase, microsomes). Each assay was repeated at least three times with similar results.

**Figure 9:**
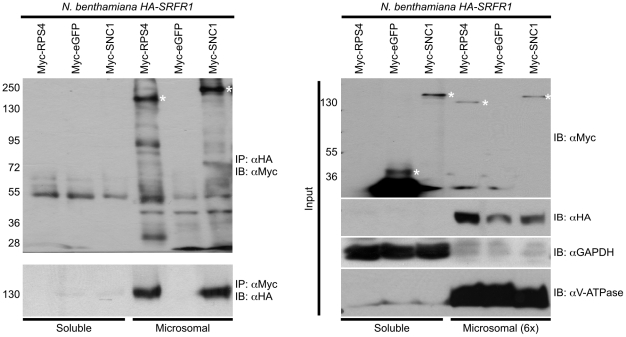
SRFR1 interacts with RPS4 and SNC1 in the microsomal fraction. Myc-RPS4, Myc-eGFP or Myc-SNC1 were transiently expressed via *Agrobacterium*-mediated transient expression in *N. benthamiana* transgenic lines 6-4 and/or 7-1 expressing HA-SRFR1. Immunoprecipitation (IP) analysis was performed on soluble and microsomal fractions with the indicated antibodies. The immunoprecipitates were immunoblotted (IB) with the indicated antibodies (left panel). The right panel shows the corresponding protein expression levels in the input fractions for immunoprecipitation analyses. In the right panel, the microsomal fraction is 6-fold enriched compared to the soluble fraction. Molecular weight of protein standards (in kD) are shown on the left of the panels. Asterisks denote the expected sizes of the Myc-tagged proteins. The degree of soluble and microsomal fraction enrichment are shown by IB analyses with anti-GAPDH and anti-V-ATPase antibodies. The assay was repeated three times with similar results.

We observed improved and reproducible HA-SRFR1 expression in the stable transgenic *N. benthamiana* lines. As in Arabidopsis, HA-SRFR1 localized to the microsomal and nuclear fractions in *N. benthamiana* (Figure 8A). A previous study showed that RPS4 was predominantly localized to microsomes [36]. Immunoblot assays of Myc-SNC1 transiently expressed in *N. benthamiana* suggested that SNC1 was mainly a soluble cytoplasmic protein, although a sizeable portion accumulated in the microsomal fractions (Figure 8B). We also detected some SNC1 in the nuclear fraction (Figure 8B). We tested for SRFR1 interaction with SNC1 and RPS4 by transiently expressing Myc-SNC1, Myc-RPS4 or Myc-eGFP as a negative control in transgenic HA-SRFR1 *N. benthamiana* plants. Co-immunoprecipitation analysis on protein isolated 48 h after infiltration of *Agrobacterium tumefaciens* strains showed that SRFR1 interacted with both SNC1 and RPS4 in the microsomal fraction (Figure 9). No significant interaction between SRFR1 and SNC1 was detected in the soluble fraction, even though SNC1 was detected in this fraction. No interaction with eGFP was detected in either fraction (Figure 9). As an additional control, we probed SRFR1 co-immunoprecipitated samples for the presence of GAPDH and V-ATPase. Neither protein was co-immunoprecipitated with SRFR1 (Figure S6A and S6B), indicating that the interactions of SRFR1 with SNC1 and RPS4 are specific.

## Discussion

*SRFR1* encodes a novel tetratricopeptide repeat (TPR)-containing protein that is conserved between plants and other eukaryotes [18]. Based on limited sequence similarity with the *Saccharomyces cerevisiae* Ssn6 and the animal OGT proteins, SRFR1 was originally proposed to function as a transcriptional repressor, perhaps with defense genes as its target. Consistent with this hypothesis, resting mRNA levels of several defense genes were slightly higher in *srfr1-1* and *srfr1-2* than in wild-type RLD [29]. This general priming of the defense system made it unlikely that SRFR1 function is limited to defenses triggered by AvrRps4. However, no alteration of resistance to DC3000 strains that trigger resistance via the *R* genes *RPM1* or *RPS2* were detected. The original analysis did not include a second TNL gene, which may be significant since TNL genes like *RPS4* signal through *EDS1*, while coiled-coil (CC)-NBS-LRR genes like *RPS2* and *RPM1* require *NDR1* [17]. With the cloning of *RPS6*, a second Arabidopsis TNL bacterial *R* gene that mediates resistance via *EDS1* to the *P. syringae* pv. syringae effector HopA1 [12], [39], we found that *rps6-1 srfr1-1* double mutants were more resistant to DC3000(*hopA1*) than the *rps6-1* single mutant [39].

### Mutations in *SRFR1* activate *SNC1*

Here we extend our analysis to the Col-0 specific TNL *R*-like gene *SNC1* and show that mutations in *SRFR1* activate *SNC1*. *SNC1* was originally identified based on an autoactivated allele that led to constitutive expression of *PR1* [23]. Subsequently, it was shown that perturbation of wild-type *SNC1* expression readily leads to autoactivation [24], [27], [40]. Our finding that *SNC1* is activated in *srfr1* mutants is reminiscent of the *bon1/cpn1* phenotype [25]–[27]. How the absence of BON1 leads to SNC1 activation is not known. In particular, it is not known if sub-pools of BON1 and SNC1 reside in the same protein complex.

Together, our data show that mutations in *SRFR1* impact three resistance specificities, namely AvrRps4-, HopA1- and SNC1-triggered immunity. The impact of *srfr1* mutations on SNC1 is novel, given that previously we observed effects of *SRFR1* mutations only in the absence of the *R* genes *RPS4* or *RPS6*. *SNC1* is therefore the first TNL gene for which a genetically direct negative regulation by *SRFR1* could be shown. Whether this is also mechanistically direct remains to be determined. Consistent with the proposed function of SRFR1 as a transcriptional repressor, we found increased mRNA levels for *SNC1*, *RPP4* and At4g16950 in *srfr1-4* plants. This altered expression level was not an indirect effect of *SNC1* activation, since *RPP4* and At4g18950 were also upregulated in the *srfr1-4 snc1-11* double mutant. Because these members of the *SNC1* locus were previously shown to be co-regulated with *SNC1* [31] and because changes in *SNC1* expression levels have been shown to cause autoactivation of SNC1 [24], we propose that mutations in SRFR1 lead to misregulated expression of *SNC1*, which in turn activates constitutive expression of an enhanced defense phenotype.

### Cross-talk between RPS4- and SNC1-mediated resistance and interactions with SRFR1

The genetic connection of *SNC1* and *RPS4* via *SRFR1* was measurable as cross-talk between these resistance pathways in disease assays under specific environmental conditions. Because it had been convincingly shown that the Col-0 *rps4-2* mutant was not fully susceptible to DC3000(*avrRps4*) [36], while we observed complete susceptibility, we tested whether environmental conditions had an influence on the Col-0 phenotypic response to DC3000(*avrRps4*). Surprisingly, we found that a mere 2°C difference in temperature changed the phenotype of *rps4-2* from almost completely resistant to DC3000(*avrRps4*) to fully susceptible. Other environmental factors that are likely to impact this response are humidity [26], with drier conditions favoring resistance, and light intensity. Because *cis* or second-site mutants with activated *SNC1* have a well-described conditional phenotype influenced by temperature and humidity, we tested whether the partial phenotype of *rps4-2* is influenced by *SNC1*.

Indeed, we were able to measure a synergistic effect of mutations in *RPS4* and *SNC1* on susceptibility to DC3000(*avrRps4*) at 22°C. In addition, in the accessions RLD and Ws-0 that do not have *SNC1*, mutations in *RPS4* result in susceptibility to DC3000(*avrRps4*) that is not influenced by changes in temperature in the range investigated here. *SNC1* was originally identified in a screen for mutants with constitutively activated defenses, and to date no cognate avirulence gene has been identified. Nevertheless, some suppressor mutants of the constitutive *snc1-1* phenotype such as *mos7* also impact effector-triggered immunity [41]. Our finding that SNC1 contributes to AvrRps4-triggered immunity further indicates that *SNC1* can be considered a *bona fide R* gene.

Conceptually, cross-talk between resistance pathways can occur if an effector protein has more than one target, or if R proteins guard a common target. The former seems to be the case for RPM1 and TAO1, which additively contribute to full resistance to DC3000(*avrB*) [42]. In contrast, AvrRpm1 induced measurable defenses in *rpm1* plants that were dependent on *RPS2*, presumably because both RPM1 and RPS2 guard RIN4, a protein that is the target for both AvrRpm1 and AvrRpt2 [19]. As a first step to distinguish between these models, we tested whether SNC1 and RPS4 co-localize with a common protein. Given the regulatory function of SRFR1 on SNC1 and on AvrRps4-triggered resistance, we speculated that SRFR1 might be such a common protein. Interestingly, the microsomal pool of SRFR1 was found to be in a complex with SNC1.

Transiently expressed GFP-SRFR1 in *N. benthamiana* localized to the nucleus and cytoplasm [18]. The cytoplasmic localization was punctate. Here, further analysis of the cytoplasmic pool showed that most SRFR1 localized to the microsomal cytoplasmic fraction, and very little was soluble. Because the majority of SNC1 was in the soluble cytoplasmic pool, it was not possible to determine whether the microsomal pool of SNC1 diminishes in the absence of SRFR1. In addition, the native *N. benthamiana* pool of SRFR1 may be sufficient to localize some proportion of SNC1 to microsomes. Most likely, SNC1 is in a higher-order complex with SRFR1 in a microsomal compartment of unknown identity. Interestingly, we found that RPS4 also interacted with SRFR1 in the same cell fraction. This suggests that perhaps additional R proteins localize to a common complex. The localization of SRFR1 and interactions with RPS4 and SNC1 are reminiscent of CRT1 [43]. However, the functions of CRT1 and SRFR1 likely differ, because mutations in *CRT1* compromise, not enhance, effector-triggered immunity.

Because mutations in *SRFR1* lead to increased, not decreased resistance, we do not propose that SRFR1 is analogous to RIN4 as the guardee of RPS4 or SNC1, since deletion of a guardee should prevent recognition of the specific effector that targets the guardee. The function of guardee for SNC1 may be fulfilled by BON1 [44], although BON1 is localized to the plasma membrane [25] and to our knowledge it has not been determined whether BON1 interacts with SNC1. Also, because no cognate effector is known for SNC1 and because deletion of BON1 leads to autoactivation of SNC1, it is difficult to quantify the effects of *BON1* mutations on disease resistance and susceptibility. Interestingly, we consistently observed a more severe growth phenotype of *srfr1-4* plants compared to *bon1-1* plants, yet the *srfr1-4* growth phenotype is completely reversed by *snc1-11*. Apart from negatively regulating the activation of SNC1, SRFR1 most likely regulates additional R proteins. Because of positive feed-back, all these pathways may be turned on once SNC1 is activated. While in *bon1-1* plants SRFR1 is still present to downregulate these other R proteins, this is not the case in *srfr1-4* plants. Therefore, this observation is suggestive of a broad and central function of SRFR1 in downregulating R protein output.

It is currently unknown where in the cell the recognition of AvrRps4 by RPS4 occurs. Several plant R proteins, including RPS4, have been shown to function in the nucleus to trigger immunity [36], [45]. Because the cytoplasmic pool of these R proteins predominates over the nuclear pool, it is difficult to establish whether R proteins translocate to the nucleus upon effector perception, or continuously cycle between the cytoplasmic and nuclear compartment. We also detected a low amount of SNC1 in the nucleus, whereas the autoactivated mutant snc1-1 protein appears to accumulate to higher levels in the nucleus [41]. It was also found that snc1-1 needed to be in the nucleus to cause a stunted phenotype [41], and that temperature modulated the localization of snc1-1 [46]. Interestingly, a balanced partitioning of EDS1 between the cytoplasm and nucleus was recently shown to be required for full EDS1-mediated resistance [47], indicating that immune regulatory proteins may have coordinated cytoplasmic and nuclear functions during the immune response.

Here we found that SRFR1 interacts with RPS4 and SNC1 in the cytoplasm, and also that mutations in *SRFR1* alter the expression of defense genes independent of a *snc1* phenotype. Because of the low amount of RPS4 [36], SRFR1 and SNC1 protein in the nucleus, so far we have not been able to ascertain whether they also interact in the nucleus. However, our results seem to suggest that at resting state, the majority of SRFR1, RPS4 and SNC1 protein is extra-nuclear localized and forms a complex in the microsomal fraction. SRFR1 may therefore negatively regulate RPS4 and SNC1 translocation to the nucleus. We propose that a second point of regulation is in the nucleus, where SRFR1 may negatively regulate the transcriptional reprogramming upon pathogen perception. More detailed analyses before and during a defense response are required to substantiate these hypotheses.

### Genetics of *srfr1*-mediated resistance revisited

The genetics of enhanced resistance in RLD *srfr1* mutants were originally interpreted to signify that an additional specific *R* gene is required for resistance [15]. In the mapping crosses *rps4-1*×*srfr1-1* and *rps4-1*×*srfr1-2*, resistant F2 plants were identified in the ratio 13 susceptible to 3 resistant, consistent with segregation of a recessive locus (*srfr1*) and a dominant locus that was proposed to be a second specific *R* gene with weak recognition of AvrRps4 [15]. In light of the results presented here, we needed to reinterpret these results. Retesting our mapping population provided evidence for severely stunted plants at the expected ratio of one in 16 stunted plants. These would be double recessives (*srfr1* and wild-type *SNC1*) and would have been lost from our usual phenotypic analysis because of preferential retention of vigorously growing seedlings after planting for disease assays. Upon reinspection, the segregation of resistant plants in the two mapping populations was indeed statistically consistent with the segregation of a single recessive locus (*srfr1*) in a population where 1/16th of the population (genotype *srfr1/srfr1 SNC1/SNC1*) that would have been expected to be resistant was eliminated from consideration. In addition, in both mapping populations we had noticed an apparent suppression of recombination along chromosome 4 in retained plants [15], which is consistent with the fact that both *SRFR1* and *SNC1* are located on chromosome 4. At the same time, we show here that the original model for resistance in *srfr1* mutants mediated by other *R* genes with weaker recognition of AvrRps4 is still valid because cross-talk between *R* genes exists in response to AvrRps4. However, we now consider it unlikely that one single additional *R* gene is responsible for resistance in *srfr1* mutants.

In conclusion, our data contribute to evidence for extensive cross-talk between at least three TNL pathways that converge on SRFR1, indicating that SRFR1 perhaps has a central function in regulating the output of additional TNL proteins. The present data also allow us to propose more directly that SRFR1 negatively regulates R proteins or *R* gene expression. While models for SRFR1 so far have focused on a nuclear-localized transcriptional repressor function [18], the data here suggest that SRFR1 also has a function in the cytoplasm. Consistent with this, Li and co-workers recently showed that SRFR1 interacts with SGT1 in the cytoplasm [22]. Whether SRFR1 is merely an accessory protein in a cytoplasmic “resistasome” or has regulatory functions and migrates to the nucleus remains to be established. Nevertheless, our data highlight molecular architecture aspects of a subset of TNL-mediated resistance pathways that will allow further mechanistic insight into the function of TNL R proteins. The cross-talk evident from our results also means that any reports of constitutive resistance phenotypes in Col-0 need to consider the possible involvement of *SNC1*.

## Materials and Methods

### Plant lines

The *srfr1-4* line (SAIL_412_E08) from the Syngenta Arabidopsis Insertion Library [21] was obtained from the Arabidopsis Biological Resource Center. The T-DNA insertion site in *srfr1-4* in the second intron was determined by sequencing and was found to be upstream of the insertion site suggested by raw flanking sequence from the T-DNA Express website (http://signal.salk.edu/cgi-bin/tdnaexpress). *rps4-2* (SALK_057697) was isolated from the Salk T-DNA knockout lines [20]. *snc1-11* (SALK_047058) and *bon1-1* were a kind gift from Jian Hua (Cornell University). Using *snc1-11* as a recipient, *srfr1-4 snc1-11* and *rps4-2 snc1-11* double homozygous mutants were generated. The mutant lines *rps4-21*, *rrs1-1* and *rps4-2 rrs1-1* in the Ws-0 background were kindly provided by Yoshihiro Narusaka (Research Institute for Biological Sciences, Japan). The mapping populations generated by crossing *srfr1-1* or *srfr1-2* to *rps4-1* (SAIL_519_B09) were described previously [15].

Complemented *srfr1-4* transgenic lines were generated by transforming *srfr1-4* with pSHK102, a genomic *SRFR1* clone in vector pCAMBIA2300 [18], using the floral dip method [48]. Single locus transgenic lines homozygous for the transgenic copy of wild-type *SRFR1* were selected by scoring for kanamycin resistance, the selectable marker of pCAMBIA2300 (the selectable marker for SAIL lines is BASTA). Among these homozygous lines, those with at least one copy of the *srfr1-4* allele were selected by genotyping and propagated to the next generation. Lines homozygous for both the *SRFR1* transgene and the *srfr1-4* allele were identified as those where *srfr1-4* did not segregate in the next generation. *SNC1* was mapped by genotyping stunted plants in the F2 generation from the cross RLD×*srfr1-4* using SSLP and CAPS markers [49], [50].

### Plant growth and *in planta* bacterial growth curve assays

Unless otherwise noted, Arabidopsis plants used in this study were grown in E-7/2 reach-in growth chambers (Controlled Environments Ltd., Winnipeg, Manitoba, Canada) under an 8 h light/16 h dark cycle at 24°C and 22°C, with 70% relative humidity and a light intensity of 90–140 µmol photons m^−2^ s^−1^. Virulent *Pseudomonas syringae* pv. tomato strain DC3000 containing the empty vector (ev) pVSP61 or DC3000 expressing *avrRps4* from plasmid pVSP61 was grown as described previously [16]. To generate DC3000 *hrcC^−^*(ev), pVSP61 was mobilized into the recipient DC3000 *hrcC* ^−^ mutant by triparental mating using the helper plasmid pRK2013. *In planta* bacterial growth assays were performed by syringe infiltration. Leaves of 4-week old plants were infiltrated with bacterial suspensions of 5×10^4^ cfu/mL. Leaf discs with a total area of 0.5 cm^2^ per sample were ground in 10 mM MgCl_2_, and solutions were plated in serial dilutions on selective medium in triplicate at the indicated time points. Statistical comparison of bacterial growth was tested using a two-tailed Student's t-test.

### Transcript profiling, reverse transcription PCR and RACE

Quantitative reverse transcription PCR was performed as described previously [18]. Briefly, total RNA was extracted from the indicated plant lines using TRIZOL (Invitrogen, Carlsbad, CA, USA). For RT-PCR experiments, cDNA was synthesized from 2 µg of total RNA using an oligo(dT)15 primer and Moloney murine leukemia virus (MMLV) reverse transcriptase (Promega, Madison, WI, USA) following the manufacturer's protocol. Quantitative real-time reverse transcription PCR (qPCR) was performed with SYBR GREEN PCR Master Mix and an ABI 7500 system (Applied Biosystems, Warrington, UK) according to the manufacturer's instructions. The levels of transcripts were normalized using *SAND* gene (At2g28390) mRNA levels as an internal standard. These experiments were performed at least twice with similar results. Semi-quantitative RT-PCR was performed from total RNA extracted from Col-0 and *srfr1-4*. Equivalent amounts of cDNA from both samples were used to detect *PR1*, *PR2* and *PDF1.2*. *ACTIN2* (At3g18780) was used as an internal control. Table S1 lists the oligonucleotide primer sequences used in qPCR and semi-quantitative RT-PCR.

To determine the *SNC1* cDNA sequence from RLD and Col-0, the 3′-RACE procedure (Invitrogen, Carlsbad, CA, USA) and RT-PCR (see above) were performed as described previously [19]. PCR products were ligated into the pGEM-T Easy vector (Promega) for sequencing. See Table S1 for oligonucleotide primer sequences used in these experiments.

### Molecular cloning and generation of transgenic *N. benthamiana* plants

All clones were verified by sequencing. To generate epitope-tagged *SNC1* constructs, genomic *SNC1* DNA including introns was amplified by PCR from Col-0 using SNC1 GATE primers listed in Table S1. *In vitro* BP Clonase recombination reactions were carried out to insert the PCR product into the pDONR201 entry vector according to the manufacturer's instructions (Invitrogen). LR reactions were performed to recombine the entry clones into GATEWAY-compatible destination vectors. Using BP and LR reactions, we constructed *Myc-gSNC1* with six Myc tags under the control of the cauliflower mosaic virus 35S promoter. Similarly, *Myc-gRPS4* was generated by amplifying the genomic fragment of *RPS4* from the *FLAG-gRPS4* construct [51] using the primers RPS4 FOR and RPS4 REV (Table S1).

To construct the binary vector expressing genomic HA-tagged *SRFR1* from its native promoter (*HA-gSRFR1*), independent PCR reactions were performed with the primer combinations HA-SRFR1 FOR/gSRFR1 XbaI REV and pCAMBIA PmeI FOR/HA-SRFR1 REV using the template pSHK102 [18]. The PCR products were mixed and used for overlap PCR with the pCAMBIA Pme I FOR/gSRFR1 XbaI REV primers. The 2.2 kb PCR product was digested with PmeI and XbaI and used for replacing the PmeI-XbaI fragment of pSHK102. The resulting binary vector was electroporated into *Agrobacterium tumefaciens* strain C58C1. Transgenic *N. benthamiana* plants expressing *HA-gSRFR1* from the Arabidopsis native promoter were generated by stable *Agrobacterium*-mediated transformation as previously described [52]. Transgenic plants were selected on media containing 100 µg/ml kanamycin.

### Protein fractionation and immunoblot analysis

Microsomal and soluble fractions were prepared according to published procedures [53]. Briefly, plant materials were ground in buffer H (50 mM HEPES, pH 7.5, 250 mM sucrose, 15 mM EDTA, 5% glycerol, 0.5% polyvinylpyrrolidone) containing 3 mM DTT and 1×protease cocktail inhibitors (Sigma, St. Louis, MO). The extracts were filtered through two layers of miracloth pre-wetted with buffer H and centrifuged at 2000×g for 15 min at 4°C. The supernatant consisting of the cytoplasmic fraction was further subjected to ultracentrifugation at 100,000×g to separate the soluble and microsomal (pellet) fractions. The pellet was resuspended in buffer H. Nuclear extracts were prepared using the CelLytic™ PN Isolation/Extraction Kit (Sigma) following the manufacturer's instructions. Total protein concentrations of fractions were determined by Bradford assays with BSA as standard. Extracts were normalized to 1 µg/ml with buffer H. For co-immunoprecipitation assays, the nonionic detergent Igepal CA-630 (Sigma) was added to 0.2% and 1% final concentration to the soluble and microsomal fractions, respectively. The extracts were incubated overnight with 20 µl of anti-HA or anti-Myc agarose beads (Sigma). The beads were washed three times with buffer H containing 0.2% Igepal CA-630. The immunoprecipitates were analyzed by immunoblot assays with anti-Myc-HRP (Santa Cruz Biotechnology) or anti-HA-HRP (Roche) antibodies. The degree of enrichment in cellular fractionation was determined by immunoblot analyses with anti-GAPDH (Genscript, Piscataway, NJ), anti-V-ATPase (Agrisera, Vännäs, Sweden), anti-histone H3 (Abcam, Cambridge, MA) and anti-RNA pol I (Agrisera) antibodies.

### TAIR accession numbers

*SNC1*: At4g16890; *SRFR1*: At4g37460; *RPS4*: At5g45250; *RPP4*: At4g16860; *NPR1*: At1g64280; *EDS1*: At3g48090; *PAD4*: At3g52430; *SID2*: At1g74710; *PR1*: At2g14610; *PR2*: At3g57260; *PDF1.2*: At5g44420; *SAND*: At2g28390; *ACTIN2*: At3g18780.

## Supporting Information

Table S1 PCR primers used in this study.
(0.07 MB PDF)

Figure S1 RT-PCR analysis of *SRFR1* transcripts in Col-0 and *srfr1-4*. (Top) Diagram of the *SRFR1* gene structure. The T-DNA insertion site in the second intron, verified by sequencing, is indicated with a triangle. Locations of primers used for PCR after reverse transcription are indicated by arrows. (Bottom) Ethidium bromide-stained gel showing PCR products obtained with the indicated primer pairs and RNA isolated from Col-0 and *srfr1-4*. *ACTIN2* was used as an internal standard to indicate equal amount of RNA used in RT-PCR.
(0.04 MB PDF)

Figure S2 Enhanced basal defenses and expression of defense genes in *srfr1-4* plants is abolished at 28°C. (A) *In planta* bacterial growth was measured in Col-0, *srfr1-4*, *snc1-11* and *srfr1-4 snc1-11* grown at 28°C on day 0 (blue bars) and day 3 (purple bars) after inoculation of DC3000 (top) and DC3000(*avrRps4*) (bottom) at 5×10^4^ cfu/ml. Values represent averages of cfu/cm^2^ leaf tissue from triplicate samples, and error bars denote standard deviation. This experiment was repeated once with similar results. (B) *SNC1* (top) and *PR1* (bottom) transcript levels were measured by qPCR in Col-0, *srfr1-4*, *snc1-11* and *srfr1-4 snc1-11* grown at 28°C, and were normalized using *SAND* gene (At2g28390) mRNA levels as an internal control. Note difference in scale compared to Figure 6. Values represent averages from six biological replicates, and error bars denote standard deviation.
(0.21 MB PDF)

Figure S3 Upregulation of *R* gene transcripts is not limited to TNL genes in Col-0. *RPM1* and *RPS2* transcript levels were quantified in Col-0 (blue bars), *srfr1-4* (purple), *snc1-11* (yellow) and *srfr1-4 snc1-11* (light blue) plants grown at 22°C (A) or 24°C (B), or in RLD (blue bars) and *srfr1-1* (purple) plants grown at 24°C (C). Transcript levels were normalized using *SAND* gene (At2g28390) mRNA levels as an internal control. Values represent averages from four (A and B) and five (C) biological replicates, and error bars denote standard deviation.
(0.20 MB PDF)

Figure S4 Mutants in the Ws-0 background do not show changes in susceptibility to DC3000(*avrRps4*) between 22°C and 24°C. *In planta* bacterial growth was measured in Ws-0, *rps4-21*, *rrs1-1* and *rps4-21 rrs1-1* on day 0 (blue bars) and day 3 (purple bars) after inoculation of DC3000 (top) and DC3000(*avrRps4*) (bottom) at 5×10^4^ cfu/ml at 22°C (A) and 24°C (B). Values represent averages of cfu/cm^2^ leaf tissue from triplicate samples, and error bars denote standard deviation. Values labeled with different letters show significant differences on day 3 as determined by the Student's t-test (*P*<0.05, n = 3). This experiment was repeated once with similar results.
(0.23 MB PDF)

Figure S5 HA-SRFR1 localizes to microsomes and nuclei of transgenic *srfr1-4* plants. (A) Trangenic *srfr1-4* plants expressing genomic *HA-SRFR1* from its native promoter show complete reversal of the stunted phenotype (left panel). Total proteins were extracted from mature leaves of Columbia (Col-0) and two *srfr1-4* transgenic lines (1 and 2) expressing genomic *HA-SRFR1* driven by the native promoter (right panel). The extracts were immunoblotted with anti-HA antibodies. The coomassie-stained blot is shown below to indicate equal loading. (B) Total proteins from Col-0 and transgenic *srfr1-4 HA-SRFR1* line 1 (*s1-4 HS1*) were fractionated into soluble, microsomal and nuclear fractions. Immunoblot analyses were performed with anti-HA to detect HA-SRFR1, and with antibodies specific to organelle markers to determine fraction enrichment (anti-RNA Pol I subunit, nucleus; anti-V-ATPase, microsomes; and anti-GAPDH, cytoplasmic soluble). The microsomal and nuclear extracts are 6 and 14 times concentrated, respectively, compared to the soluble fraction.
(0.70 MB PDF)

Figure S6 SRFR1 does not interact with GAPDH or V-ATPase. (A) Immunoprecipitates with anti-HA antibodies of the soluble fraction shown in Figure 9 were immunoblotted with anti-GAPDH antibodies. The last lane of the panel contains soluble extracts from transient expression of Myc-eGFP in transgenic *N. benthamiana* plants expressing HA-SRFR1. (B) Immunoprecipitates with anti-HA antibodies of the microsomal fraction shown in Figure 9 were immunoblotted with anti-V-ATPase antibodies. The last lane of the panel contains microsomal extracts from transient expression of Myc-eGFP in transgenic *N. benthamiana* plants expressing HA-SRFR1.
(0.28 MB PDF)
