# Supplementary material for: The Arabidopsis Resistance-Like Gene SNC1 Is Activated by Mutations in SRFR1 and Contributes to Resistance to the Bacterial Effector AvrRps4
Source: PLoS Pathog. 2010 Nov 4;6(11):e1001172. doi: 10.1371/journal.ppat.1001172 (PMC2973837; doi:10.1371/journal.ppat.1001172)
Supplement: Table S1 — PCR primers used in this study. (0.07 MB PDF) [file ppat.1001172.s001.pdf]

Table S1. PCR primers used in this study

Primer	Sequences (5'-3')	Purpose
37460-18	TCTCCACTGTACTAATTTCCCT	*SRFR1* genotyping
37460-R	ACTAATTCCGCAACGTGCCT	*SRFR1* genotyping
LB3	TAGCATCTGAATTTCATAACCAATCT CGATACAC	*srfr1-4* genotyping (with 37460-R)
ColB1	ATATGGAGATAGCTTCTTCTTCTG	*SNC1* genotyping
ColB4	AAGATCATATCCGGCCATAACTG	*SNC1* genotyping
JMLB2	TTGGGTGATGGTTCACGTAGTGGG	*snc1-11* genotyping (with ColB1)
SAND F	AACTCTATGCAGCATTTGATCCACT	qPCR
SAND R	TGATTGCATATCTTTATCGCCATC	qPCR
SRFR1F	CTGG`ATATGCCTCACTAGAAG	qPCR
SRFR1R	CACTGGGTCACAAGGCTCTG	qPCR
SNC1 F	GCCGGATATGATCTTCGGAA	qPCR
SNC1 R	CGGCAAGCTCTTCAATCATGG	qPCR
RPP4 F	GAAGGCACTCAAGGCCTCATT	qPCR
RPP4 R	GACAATAATCCCACCATAGCCTTT	qPCR
16950 F	TGGGTGCAAGCTCTCACAGA	qPCR
16950 R	TCATTAGGCCCGTTCAGAAGA	qPCR
RPS2 F2	CTTGAGAACTAGGGATCTGCC	qPCR
RPS2 R2	TCACAAATTGCCCCAGAGTT	qPCR
RPM1 F2	AGCTTTATGTCAGGGCTTGT	qPCR
RPM1 R2	CAGTTAGTAGAACGCATTCGC	qPCR
NPR1 F	GTCGGTGAGA CTCTTGCCTC	qPCR
NPR1 R	CGAAGAGCGAAACTATATGACG	qPCR
EDS1 F	GACGGGGAAGTAGATGAGAAG	qPCR
EDS1 R	TCATCCATCATACGCTCACG	qPCR
PAD4 F	GAGGAGATCTTTGTTACGGG	qPCR
PAD4 R	TCGCCTCCCACACACTATAA	qPCR
SID2 F	GCAACA ACATCTCTAC AGGCG	qPCR
SID2 R	AGAACCCCTTATCCCCCATA	qPCR

PR1 F1	CCCTCGAAAG CTCAAGATAG	qPCR
PR1 F2	GCAATGGAGTTTGTGGTCAC	Semi-qPCR
PR1 R	GTTCACATAATTCCCACGAGG	Semi-qPCR & qPCR
PDF1.2 F1	AAGTTGTGCGAGAAGCCAAG	qPCR
PDF1.2 R1	CCATGTTTGGCTCCTTCAAG	qPCR
PDF1.2 F2	TCATCATGGCTAAGTTTGCTTCC	Semi-qPCR
PDF1.2 R2	AATACACACGATTTAGCACC	Semi-qPCR
PR2 F	ATGCTACGGGATGCTAGGCG	Semi-qPCR
PR2 R	TCTCCGACACCACGATTTCC	Semi-qPCR
ACTIN2 F	TCGGTGGTTCCATTCTTGCT	Semi-qPCR
ACTIN2 R	GCTTTTTAAGCCTTTGATCTTGAGAG	Semi-qPCR
SNC1 GATE F1	AAAAAGCAGGCTCAATGGAGATAGC TTCTTCTTCTGG	Cloning of *gSNC1* entry
SNC1 GATE R1	AGAAAGCTGGGTAGTTACCAGAAAC AGGAAACAAGA	Cloning of *gSNC1* entry
SNC1 FB1	ATATGGAGATAGCTTCTTCTTCTG	Cloning of *cSNC1-RLD*
SNC1 R32	ACCCAAAGTCTGATTTATTCATTG	Cloning of *cSNC1-RLD*
SNC1 F17	TTTCGCCCAGAACAACTCGC	Cloning of *cSNC1-Col*
SNC1 R2	CAAACGCAGAGTTGGATAATGT	Cloning of *cSNC1-Col*
RPS4 FOR	AAAAAGCAGGCTCAATGGAGACATC ATCTATT	Cloning of *gRPS4* entry
RPS4 REV	AGAAAGCTGGGTATCAGAAATTCTT AACCGTGTG	Cloning of *gRPS4* entry
HA-SRFR1 FOR	TGTTCCAGATTATGCTAGTCTTATGG CGACGGCGACG	Cloning of *HA-gSRFR1*
HA-SRFR1 REV	TAATCTGGAACATCATAAGGATACA TTTTTTGAGCGG	Cloning of *HA-gSRFR1*
pCAMBIA Pme I FOR	CCGCCAATATATCCTGTCAAACACT	Cloning of *HA-gSRFR1*
gSRFR1 XbaI REV	GTGTATTGCTATACCTGTGCAATGC	Cloning of *HA-gSRFR1*
