# Supplementary figures and images for: The Arabidopsis Resistance-Like Gene SNC1 Is Activated by Mutations in SRFR1 and Contributes to Resistance to the Bacterial Effector AvrRps4
Source: PLoS Pathog. 2010 Nov 4;6(11):e1001172. doi: 10.1371/journal.ppat.1001172 (PMC2973837; doi:10.1371/journal.ppat.1001172)

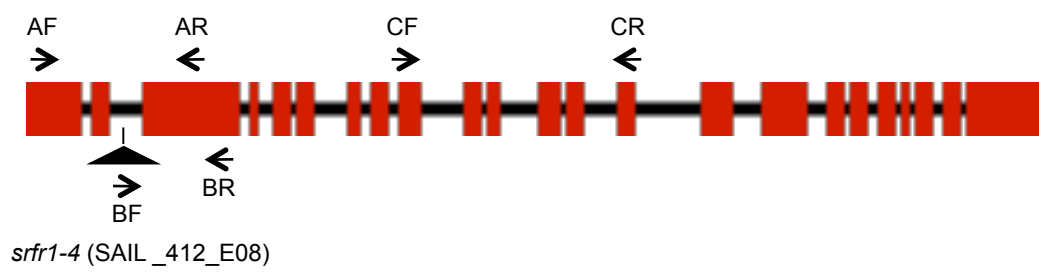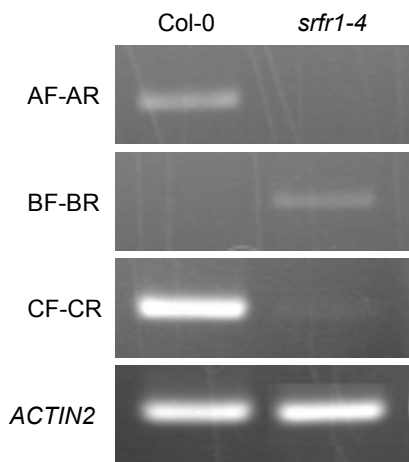

Supplement: Figure S1 — RT-PCR analysis of SRFR1 transcripts in Col-0 and srfr1-4. (Top) Diagram of the SRFR1 gene structure. The T-DNA insertion site in the second intron, verified by sequencing, is indicated with a triangle. Locations of primers used for PCR after reverse transcription are indicated by arrows. (Bottom) Ethidium bromide-stained gel showing PCR products obtained with the indicated primer pairs and RNA isolated from Col-0 and srfr1-4. ACTIN2 was used as an internal standard to indicate equal amount of RNA used in RT-PCR. (0.04 MB PDF) [file ppat.1001172.s002.pdf]

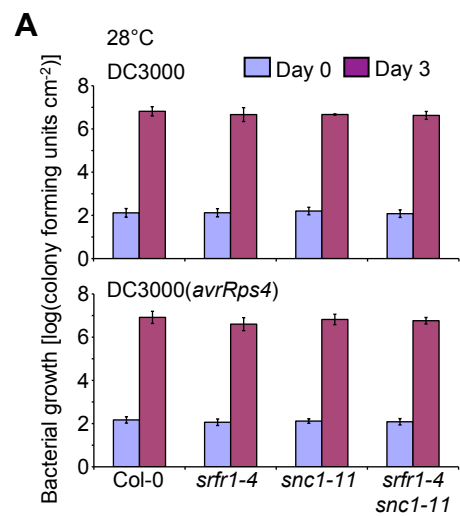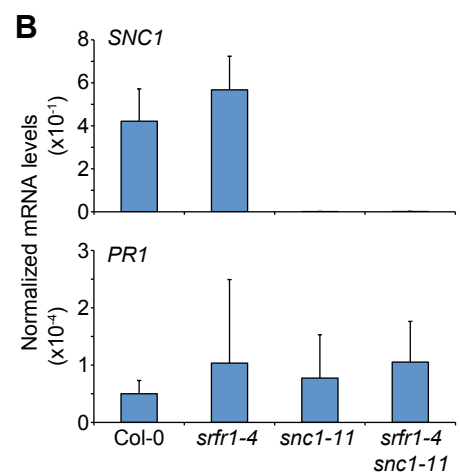

Supplement: Figure S2 — Enhanced basal defenses and expression of defense genes in srfr1-4 plants is abolished at 28°C. (A) In planta bacterial growth was measured in Col-0, srfr1-4, snc1-11 and srfr1-4 snc1-11 grown at 28°C on day 0 (blue bars) and day 3 (purple bars) after inoculation of DC3000 (top) and DC3000(avrRps4) (bottom) at 5×10^4^ cfu/ml. Values represent averages of cfu/cm2 leaf tissue from triplicate samples, and error bars denote standard deviation. This experiment was repeated once with similar results. (B) SNC1 (top) and PR1 (bottom) transcript levels were measured by qPCR in Col-0, srfr1-4, snc1-11 and srfr1-4 snc1-11 grown at 28°C, and were normalized using SAND gene (At2g28390) mRNA levels as an internal control. Note difference in scale compared to Figure 6. Values represent averages from six biological replicates, and error bars denote standard deviation. (0.21 MB PDF) [file ppat.1001172.s003.pdf]

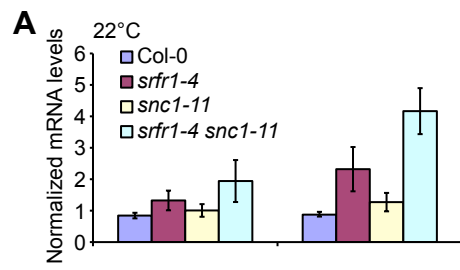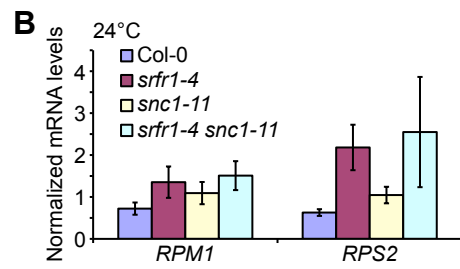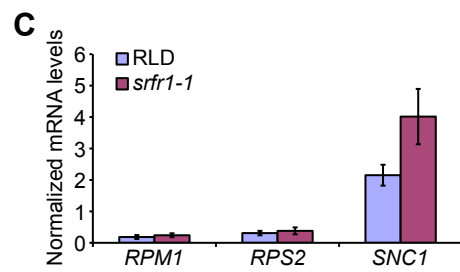

Supplement: Figure S3 — Upregulation of R gene transcripts is not limited to TNL genes in Col-0. RPM1 and RPS2 transcript levels were quantified in Col-0 (blue bars), srfr1-4 (purple), snc1-11 (yellow) and srfr1-4 snc1-11 (light blue) plants grown at 22°C (A) or 24°C (B), or in RLD (blue bars) and srfr1-1 (purple) plants grown at 24°C (C). Transcript levels were normalized using SAND gene (At2g28390) mRNA levels as an internal control. Values represent averages from four (A and B) and five (C) biological replicates, and error bars denote standard deviation. (0.20 MB PDF) [file ppat.1001172.s004.pdf]

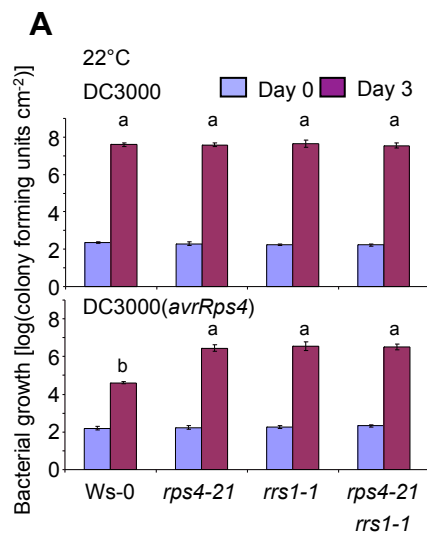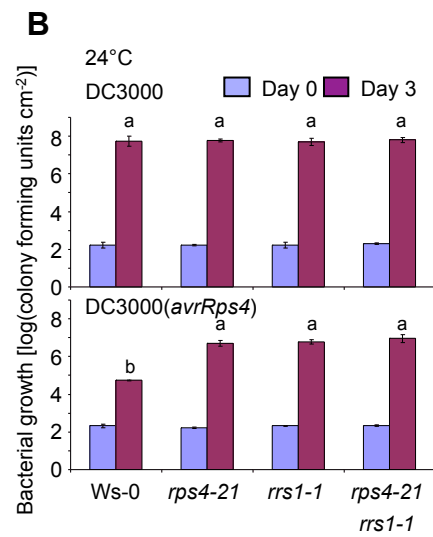

Supplement: Figure S4 — Mutants in the Ws-0 background do not show changes in susceptibility to DC3000(avrRps4) between 22°C and 24°C. In planta bacterial growth was measured in Ws-0, rps4-21, rrs1-1 and rps4-21 rrs1-1 on day 0 (blue bars) and day 3 (purple bars) after inoculation of DC3000 (top) and DC3000(avrRps4) (bottom) at 5×10^4^ cfu/ml at 22°C (A) and 24°C (B). Values represent averages of cfu/cm2 leaf tissue from triplicate samples, and error bars denote standard deviation. Values labeled with different letters show significant differences on day 3 as determined by the Student's t-test (P<0.05, n = 3). This experiment was repeated once with similar results. (0.23 MB PDF) [file ppat.1001172.s005.pdf]

**A**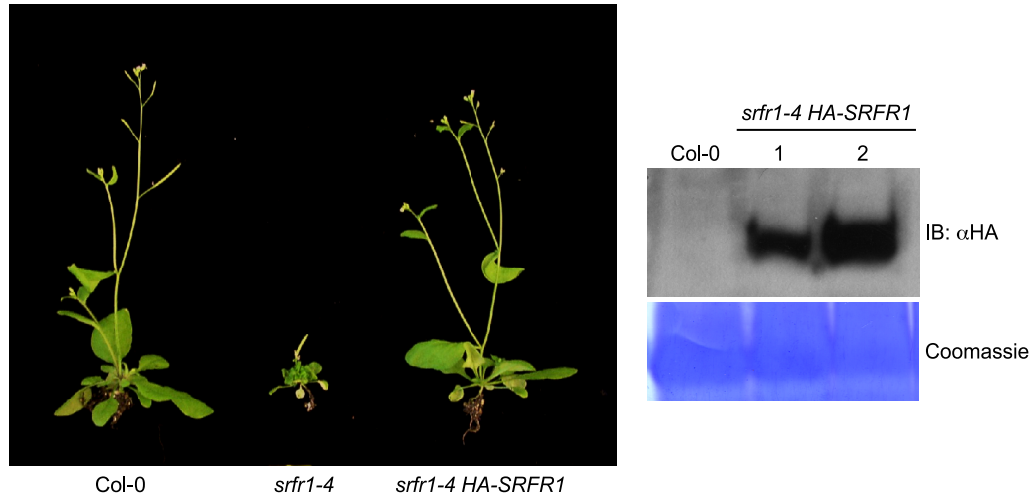**B**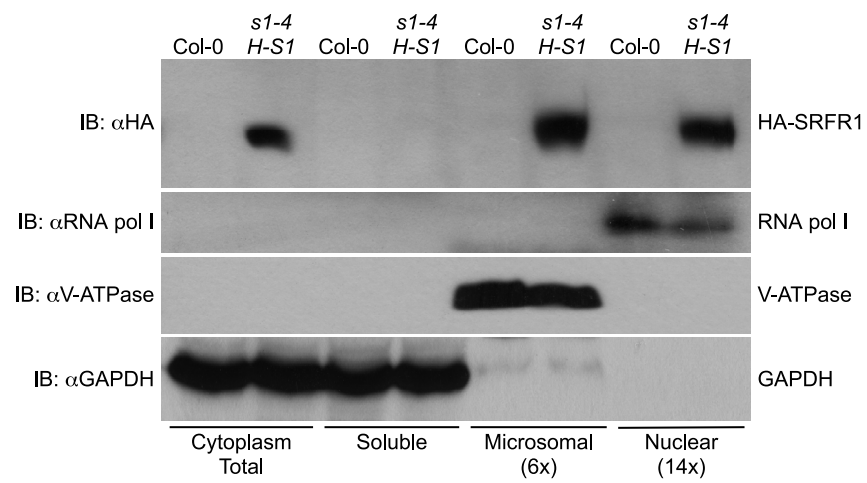

Supplement: Figure S5 — HA-SRFR1 localizes to microsomes and nuclei of transgenic srfr1-4 plants. (A) Trangenic srfr1-4 plants expressing genomic HA-SRFR1 from its native promoter show complete reversal of the stunted phenotype (left panel). Total proteins were extracted from mature leaves of Columbia (Col-0) and two srfr1-4 transgenic lines (1 and 2) expressing genomic HA-SRFR1 driven by the native promoter (right panel). The extracts were immunoblotted with anti-HA antibodies. The coomassie-stained blot is shown below to indicate equal loading. (B) Total proteins from Col-0 and transgenic srfr1-4 HA-SRFR1 line 1 (s1-4 HS1) were fractionated into soluble, microsomal and nuclear fractions. Immunoblot analyses were performed with anti-HA to detect HA-SRFR1, and with antibodies specific to organelle markers to determine fraction enrichment (anti-RNA Pol I subunit, nucleus; anti-V-ATPase, microsomes; and anti-GAPDH, cytoplasmic soluble). The microsomal and nuclear extracts are 6 and 14 times concentrated, respectively, compared to the soluble fraction. (0.70 MB PDF) [file ppat.1001172.s006.pdf]

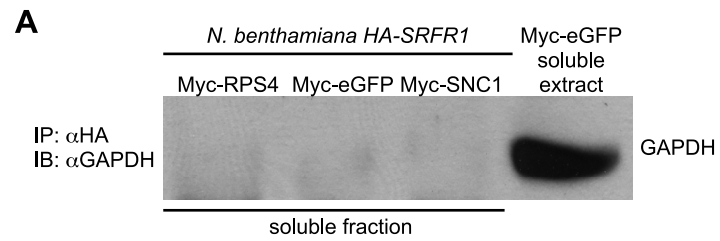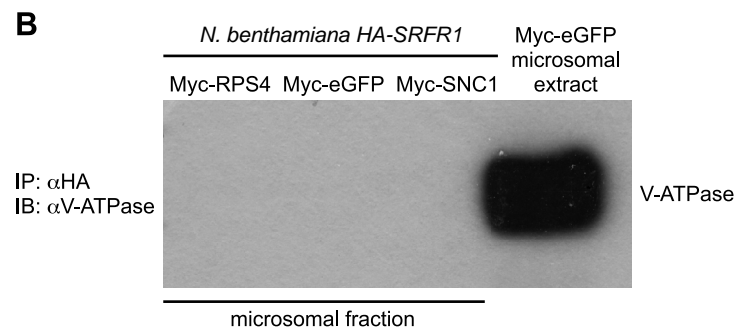

Supplement: Figure S6 — SRFR1 does not interact with GAPDH or V-ATPase. (A) Immunoprecipitates with anti-HA antibodies of the soluble fraction shown in Figure 9 were immunoblotted with anti-GAPDH antibodies. The last lane of the panel contains soluble extracts from transient expression of Myc-eGFP in transgenic N. benthamiana plants expressing HA-SRFR1. (B) Immunoprecipitates with anti-HA antibodies of the microsomal fraction shown in Figure 9 were immunoblotted with anti-V-ATPase antibodies. The last lane of the panel contains microsomal extracts from transient expression of Myc-eGFP in transgenic N. benthamiana plants expressing HA-SRFR1. (0.28 MB PDF) [file ppat.1001172.s007.pdf]
